# Endothelial-Cell-Derived Human Secretory Leukocyte Protease Inhibitor (SLPI) Protects Cardiomyocytes against Ischemia/Reperfusion Injury

**DOI:** 10.3390/biom9110678

**Published:** 2019-10-31

**Authors:** Kantapich Kongpol, Nitirut Nernpermpisooth, Eakkapote Prompunt, Sarawut Kumphune

**Affiliations:** 1 Biomedical Research Unit in Cardiovascular Sciences (BRUCS), Faculty of Allied Health Sciences, Naresuan University, Phitsanulok 65000, Thailand; kantapichk58@email.nu.ac.th (K.K.); nitirutn@nu.ac.th (N.N.); eakkabhoth@gmail.com (E.P.); 2 Graduate Program in Biomedical Sciences, Faculty of Allied Health Sciences, Naresuan University, Phitsanulok 65000, Thailand; 3 Department of Cardio-Thoracic Technology, Faculty of Allied Health Sciences, Naresuan University, Phitsanulok 65000, Thailand; 4 Department of Medical Technology, School of Allied Health Sciences, University of Phayao, Phayao 56000, Thailand; 5 Department of Medical Technology, Faculty of Allied Health Sciences, Naresuan University, Phitsanulok 65000, Thailand

**Keywords:** ischemia/reperfusion injury, anti-protease, secretory leukocyte protease inhibitor, protease inhibitor, cardioprotection, endothelial cells, cardiomyocytes

## Abstract

Vascular endothelial cell (EC)-derived factors play an important role in endothelial–cardiomyocyte crosstalk and could save cardiomyocytes (CMs) from injury. The manipulation of endothelial cells to secrete protective factors could enhance cardioprotection. Secretory leukocyte protease inhibitor (SLPI) has been known to protect the heart. The goal of this study was to evaluate the in vitro paracrine protective effect and mechanisms of EC-derived human SLPI on cardiomyocytes subjected to hypoxia/reoxygenation (H/R) injury. Stable endothelial cells overexpressing human SLPI were generated from an endothelial cell line (EA.hy926). The cytoprotective effect was determined by cell survival assay. The results showed that endothelial-derived recombinant human SLPI (rhSLPI) reduced simulated ischemia/reperfusion (I/R)-(81.75% ± 1.42% vs. 60.27% ± 2.52%, *p* < 0.05) and hypoxia/reoxygenation (H/R)-induced EC injury (83.57% ± 1.78% vs. 63.07% ± 1.93%, *p* < 0.05). Moreover, co-culture of ECs overexpressing rhSLPI with CMs at ratios 1:1 and 1:3 or treatment with conditioned medium enhanced cell viability by 10.51–16.7% (co-culture) and 15.25–20.45% (conditioned medium) by reducing intracellular reactive oxygen species (ROS) production, the Bax/Bcl-2 expression ratio, caspase-3, and caspase-8, and in preconditioned CMs by activation of p38 MAPK and Akt survival kinase. In conclusion, this study showed for the first time that EC-derived rhSLPI provided cardio-vasculoprotective effects against I/R injury as a possible alternative therapeutic strategy for cardioprotection.

## 1. Introduction

Myocardial ischemia/reperfusion (I/R) is a pathological process of cardiomyocytes involving inflammatory responses [1]. Reperfusion injury is also caused by oxidative damage which stimulates infiltration of leukocytes to the injured area. It is well known that cardiomyocytes (CMs) and endothelial cells (ECs) are two of the most abundant cardiac cell types in the heart. I/R injury affects and also harms surrounding cells, especially endothelial cells (ECs). This causes endothelial dysfunction [2,3] which aggravates CM injury and consequently results in expansion of the damage and possible death [4]. Although several reports have demonstrated that ECs are more sensitive to I/R injury than CMs [3,4,5], previous studies also suggested that ECs provide cytoprotection for CMs under hypoxia/reoxygenation (H/R) conditions [6]. Overproduction of reactive oxygen species (ROS) in I/R injury also stimulated the expression of endothelial cell adhesion molecules (CAMs) and enhanced vascular permeability [4,7], while recruiting and promoting neutrophil infiltration to the ischemic area [4,5,7]. Infiltrated leukocytes produce and secrete various protease enzymes which aggravate resident cell injury [2,8], especially in ECs and CMs. Thus, any strategy to lower oxidative stress and provide anti-protease has the potential to reduce the expansion of cardiac cell injury, reduce the severity of the outcome, and save patients’ lives.

Secretory leukocyte protease inhibitor (SLPI) is a selective peptide inhibitor for serine proteases and counteracts excessive inflammatory responses, primarily in mucosal secretions [9]. There has been no information regarding the gene expression or SLPI secretion from the vasculature, either to the blood stream or at basal sites. A previous study showed that adding recombinant human SLPI (rhSLPI) in heart preservative solution restored myocardial contraction to normal, reduced TGF-β expression, and inversely correlated with protease enzyme activity [10]. Our previous reports also demonstrated the cardioprotective effect of SLPI. Overexpression of human SLPI in rat cardiac myoblasts (H9c2) or treatment with rhSLPI in both H9c2 and adult rat ventricular myocytes (ARVMs) reduced cell death and injury from an in vitro I/R injury [11,12], as well as decreasing the infarct size [12]. Furthermore, SLPI also protected non-cardiomyocytes such as adult rat cardiac fibroblast cells (ARCFs) and human umbilical vein endothelial cells (HUVECs) from an in vitro simulated I/R (sI/R) injury [13,14]. These reports focused on the direct effect of recombinant human SLPI treatment on individual cardiac resident cells (CM, EC, CFs). No study has reported the effect of ectopic expression of SLPI in terms of cell–cell communication, especially endothelial–cardiomyocyte (EC–CM) cross talk.

Several EC-derived factors such as nitric oxide (NO), endothelin-1 (ET-1), and neuregulin-1 (NRG-1) have been reported to induce paracrine signaling to neighboring cells. One of the crucial roles of EC–CM crosstalk was highlighted: findings showed that ECs protected CMs from injury [6]. Therefore, the manipulation of EC–CM crosstalk, especially by overexpression of EC-derived cardioprotective factors, is a possible pathway to enhance the protective power of EC secretome to reduce both EC and CM damage, especially in myocardial I/R injury. Therefore, the current study hypothesized that endothelial-derived rhSLPI could prime the cardiomyocytes and enhance the cardioprotective effect on cardiac cells subjected to in vitro I/R injury. To evaluate our hypothesis, we generated endothelial-cell-specific overexpression of rhSLPI and determined the effect of rhSLPI secreted from endothelial cells on I/R-injury-induced cardiac cell death.

## 2. Materials and Methods

### 2.1. Chemicals and Reagents

Dulbecco’s Modified Eagle Medium (DMEM), fetal bovine serum (FBS), and penicillin/streptomycin were purchased from Gibco (Gibco BRL, Life Technologies, Inc., NY, USA). pCMV3-SLPI-GFPSpark tag plasmid was purchased from Sino Biological Inc. (Sino Biological Inc., Beijing, China). This plasmid is a 6848 bp vector containing cDNA of human SLPI (NM_003064.2, the NCBI reference sequence). Forty percent (*w*/*v*) polyacrylamide gel, polyvinylidenedifluoride (PVDF) membrane, and enhanced chemiluminescence (ECL) were purchased from Merck Millipore (Merck, Darmstadt, Germany). Antibodies recognizing phosphorylated-p38, total-p38, phosphorylated-Akt, total-Akt, Bax, Bcl-2, caspase-3, caspase-8, and GAPDH were purchased from Santa Cruz Biotechnology (Santa Cruz Biotechnology, Inc., Dallas, TX, USA). All other chemicals were purchased from Sigma (Sigma, St. Louis, MO, USA).

### 2.2. Cell Culture

The human umbilical vein cell line (EA.hy926) and rat embryonic cardiomyocyte cell line (H9c2) were purchased from American Type Culture Collection (ATCC, Manassas, VA, USA) as ATCC-CRL2992 and ATCC-CRL1446, respectively. Cells were cultured in Dulbecco’s Modified Eagle Medium supplemented with 10% fetal bovine serum and 5000 units/mL of penicillin/streptomycin and were maintained in a humidified atmosphere of 95% air and 5% carbon dioxide at 37 °C until grown to 70–80% confluence prior to performing experiments.

### 2.3. Overexpression and Clonal Selection of EA.hy926 Overexpressing rhSLPI

**Figure 1:**
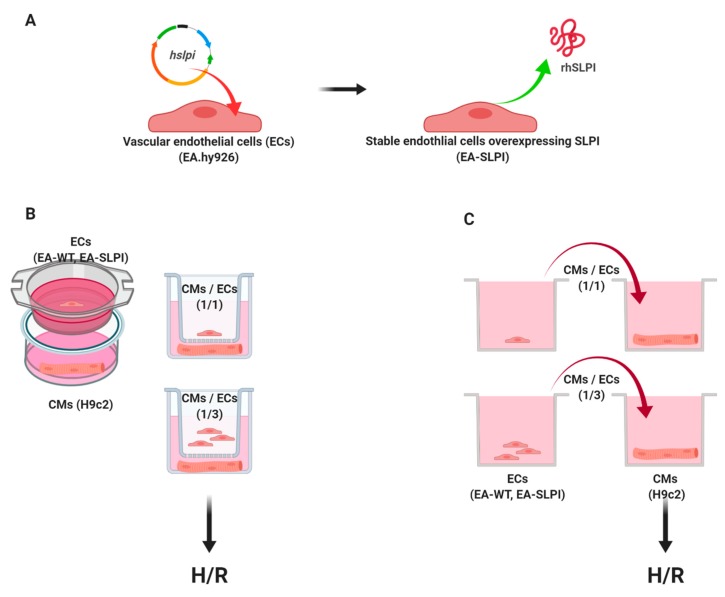
Schematic diagram of the experimental design. Vascular endothelial cells (EC) (EA.hy926) were stably transfected by a eukaryotic expression vector containing human secretory leukocyte protease inhibitor (SLPI) mRNA (*hspli*) (**A**). The stable cells overexpressing recombinant human SLPI were used for further experiments. Determination of the paracrine effect of endothelial-derived SLPI on cardiomyocyte (CM) (H9c2) subjected to hypoxia/reoxygenation (H/R) injury was performed via techniques following two different principles: co-culture between SLPI-overexpressing EA.hy926 cells and H9c2 cells by the Transwell culture system (**B**), and conditioned medium from SLPI-overexpressing EA.hy926 cells (**C**).

EA.hy926 was seeded into a 6-well plate until the cell density reached 70–80% confluence overnight prior to transfection with the pCMV3-SLPI-GFPSpark tag plasmid using a Lipofectamine^®^ 2000 (Invitrogen, Carlsbad, CA, USA) (Figure 1). After the transfection process, EA.hy926 was incubated at 37 °C in a humidified CO_2_ incubator for 24 h. Then, the medium was removed and replaced by complete medium for a further 48 h before changing to DMEM containing 100 µg/mL hygromycin B (Invitrogen, Carlsbad, CA, USA) to select positive transfectants. Cells were subjected to continuous selection in the drug and expanded from a single clonal cell until a stable overexpression cell line was established. Stable cells were cultured until they reached at least three passages before utilization in further experiments.

For SLPI-overexpressing cells (EA-SLPI), internal quality control was performed to achieve a similar level of secreted SLPI. The experiments using EA-SLPI were performed by using cells in the same passage number and an equal quantity and density of the cells. The amount of SLPI secreted from overexpressing cells was measured by ELISA before performing the experiment, which kept the SLPI level in the same range.

### 2.4. Measurements of the SLPI Level by ELISA

Human SLPI production was determined using a Quantikine^®^ Sandwich ELISA Kit purchased from R&D Systems (R&D Systems, Inc., Minneapolis, MN, USA). Briefly, 100 μL of assay diluents RD1Q and culture medium were adding on a microplate pre-coated with the SLPI-specific monoclonal antibody. Then, the solution was removed and washed with 400 μL of washing buffer three times. For detection, 200 μL of detection antibody conjugated with horseradish peroxidase (HRP) was added to the microplate and incubated at room temperature (RT) for 2 h. After incubation, the unbound detection antibody was removed and washed three times. Then, 200 μL of HRP substrates was added to the microplate and incubated at RT for 20 min in the dark. Finally, 50 μL of stop solution was added to the microplate, and the microplate color was determined using a spectrophotometer at 450 nm. The concentration of rhSLPI was proportional to the color intensity and was calculated quantitatively by comparison with the standard curve.

### 2.5. Determination of Growth Curve and Cell Viability

Cells were seeded at seeding densities of 1.5 × 10^3^ cells into 96-well cell culture plates. Cell viability was determined by MTT (3-[4,5-dimethylthiazol-2-yl]-2,5-diphenyltetrazolium bromide) assay daily for 7 days. For the MTT assay, the cultured medium was removed and replaced with 0.5 mg/mL MTT reagent. The reaction was incubated for 2 h at 37 °C. After that, MTT reagent was removed and DMSO was added to solubilize the formazan crystal. The solution was collected and the optical density (OD) was determined using a spectrophotometer at a wavelength of 490 nm, using DMSO as a blank. A growth curve was plotted between the OD and day number. The percentage of difference in reduction between control and treatment cells, under various conditions, was calculated following the formula Td = (*t*_2_ − *t*_1_) × [log(*q*_2_)/log(*q*_2_/*q*_1_)], where *q*_1_ is the quantity of the cells at the start time (1 unit/h), *q*_2_ is the quantity of the cells at the end time (1 unit/h), *t*_1_ is the starting time, and *t*_2_ is the ending time.

### 2.6. Determination of Cytoskeleton Organization

Wild-type EA.hy926 cells or SLPI-overexpressing EA.hy926 were cultured in an 8-well chamber slide. Then, cells were washed twice with PBS and fixed with 4% formaldehyde for 30 min before they were permeabilized with 0.5% Triton-X 100 for 20 min at RT. After that, cells were incubated with Tetramethylrhodamine (TRITC)-conjugated Phalloidin (Sigma, St. Louis, MO, USA) for 40 min in a dark moist chamber and subsequently washed twice with PBS. After washing, the cells were stained with DAPI (Sigma, St. Louis, MO, USA) for 20 min in the dark before observation under a fluorescent microscope.

### 2.7. Determination of Cellular Migration by Wound Healing Assay

To determine the migration ability of ECs by wound healing assay, wild-type and SLPI-overexpressing EA.hy926 monolayer scratch cells were seeded into a 6-well plate and cultured to >90% confluence [15]. Then, scratch wounding was performed using a 200 µL pipette tip. After that, cell debris and loose cells were removed by washing twice with PBS before changing to fresh completed medium. Migration was monitored for 24 h by taking photographs. Quantification of the area was calculated by ImageJ software via the following equation: [1 − (A*_t_*_=24_/A*_t_*_=0_)] × 100%, where A is the area achieved and *t* is the time point at 0 or 24 h.

### 2.8. Simulated Ischemia/Reperfusion (sI/R) Protocol

Simulated ischemia (sI) was performed following the method mentioned in previous studies [11,12]. Wild-type or SLPI-overexpressing EA.hy926 cells were seeded into a 24-well tissue culture plate at a density of 1.5 × 10^4^ cells/well and incubated with simulated ischemic basic buffer (137 mM NaCl, 3.8 mM KCl, 0.49 mM MgCl_2_, 0.9 mM CaCl_2_, 4.0 mM HEPES) containing 20 mM 2-deoxyglucose, 20 mM sodium lactate, and 1 mM sodium dithionite at pH 6.5. Cells from both groups were subjected to sI for 40 min, followed by replacement with completed medium and incubation at 37 °C, 5% CO_2_ for 24 h reperfusion (sI/R). After reperfusion, cell viability was determined by MTT assay.

### 2.9. Hypoxia/Reoxygenation (H/R) Protocol

The H/R protocol was modified from a previous study [16]. Briefly, cells were seeded into a 24-well tissue culture plate at a density of 1.5 × 10^4^ cells/well and left overnight. Then, cells were subjected to H/R using overlaying paraffin liquid on the culture media to mimic hypoxic conditions. Cells were subjected to hypoxia for 1 h and reoxygenated by replacing with completed medium for 3 h at 37 °C. After reoxygenation, cell viability was determined by MTT assay.

### 2.10. Determination of the Paracrine Effect of Endothelial-Derived SLPI on Cardiomyocyte (H9c2) Cell Injury: Co-Culture and Condition Medium Transfer

Determination of the paracrine effect of endothelial-derived SLPI on cardiomyocyte (H9c2) cell injury was performed using either indirect co-culture between SLPI-overexpressing EA.hy926 cells and H9c2 cells by the Transwell culture system or the conditioned medium from SLPI-overexpressing EA.hy926 cells (Figure 1).

Co-culture was performed using a 24-transwell permeable plate (NEST, San Diego, CA, USA) consisting of upper and lower chambers. H9c2 cells at a density of 1.5 × 10^4^ cells/well were seeded in the lower chamber. Wild-type (EA-WT) or SLPI-overexpressing EA.hy926 cells (EA-SLPI) at 1.5 × 10^4^ cells/well (CM/EC ratio of 1:1) or 4.5 × 10^4^ cells/well (CM/EC ratio of 1:3) were seeded in the upper chamber. Cells were cultured together for 48 h before being subjected to H/R (Figure 1B).

In the conditioned medium experiments, wild-type (EA-WT) or SLPI-overexpressing EA.hy926 cells (EA-SLPI) were seeded at density 1.5 × 10^4^ cells/well (for the 1CM/1EC group) or 4.5 × 10^4^ cells/well (1CM/3EC group) into 24-well tissue culture plates for 48 h. Then, the conditioned medium was collected. The H9c2 cells at were seeded at density 1.5 × 10^4^ cells/well for 24 h. Then, the H9c2 cells were incubated with conditioned medium of wild-type or SLPI-overexpressing EA.hy926 cells for 1 h prior to H/R (Figure 1C).

### 2.11. Determination of Intracellular ROS Production

The method to determine intracellular ROS production was described previously [12]. Briefly, cells were cultured with DMEM in 96-well cell culture plates. The conditioned medium was collected. Then, the cells were washed twice with PBS before incubation with DMEM containing 25 µM carboxy-H2DCFDA in a dark room for 30 min at 37 °C. After that, the carboxy-H2DCFDA was discarded, and the collected conditioned medium was restored, added back to the cells, and incubated for 24 h. For oxidative stress challenging, cells were exposed to 250 µM H_2_O_2_ for 30 min at 37 °C or subjected to sI for 40 min or hypoxia for 24 h. Intracellular ROS were determined by measuring the fluorescence intensity using an EnSpire Multimode Plate Reader (PerkinElmer, MA, USA) with excitation wavelength at 498 nm and emission wavelength at 522 nm.

### 2.12. Immunoblotting

After H/R, H9c2 cells cultured in wild-type or SLPI-overexpressing EA.hy926 media were collected for protein concentration by adding 2× SDS sample buffer containing β-mercaptoethanol as previously described [11]. Cells were scraped and transferred to a new pre-cooled microcentrifuge tube. Samples were boiled for 5 min and stored at −80 °C until required for analysis. Western blots were probed for phosphorylated p38, total-p38, phosphorylated Akt, total-Akt, Bax, Bcl-2, caspase-3, and caspase-8 at ratio 1:1000 in 1% skim milk in Tris Buffered Saline with Tween (TBST) buffer at 4 °C overnight. Secondary antibodies were either goat anti-mouse or anti-rabbit IgG antibodies conjugated to horseradish peroxidase (HRP) at ratio 1:5000. Proteins of interest were detected by chemiluminescence gel documentation. Band densities were quantified and compared to provide information on the relative abundance of the proteins of interest.

### 2.13. Statistical Analysis

Statistical analysis was performed using commercially available software (GraphPad Prism version 5, San Diego, CA, USA). All data are expressed as mean ± SEM. All comparisons were assessed for significance using an unpaired *t*-test or ANOVA, followed when appropriate by the Tukey–Kramer test. A *p*-value less than 0.05 was considered statistically significant.

## 3. Results

### 3.1. Establishment of a Stable EA.hy926 Cell Line Overexpressing rhSLPI

**Figure 2:**
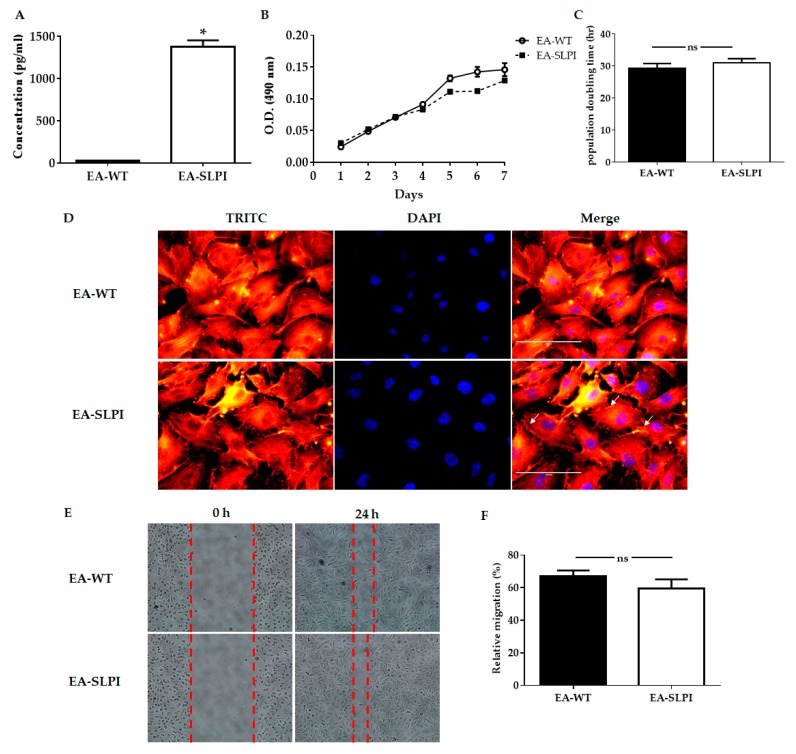
Biological characterization of SLPI-overexpressing EA.hy926 cells. The EA.hy926 cells were transfected with pCMV3-SLPI-GFPSpark tag plasmid by using lipofectamine^®^ 2000, and the level of SLPI production, growth curve, population doubling time (PDT), and morphology were determined. (**A**) The level of SLPI production in culture medium was determined by using a human SLPI ELISA kit; (**B**) Wild-type (EA-WT) and SLPI-overexpressing (EA-SLPI) EA.hy926 cells were assessed for 7 consecutive days; (**C**) Wild-type and SLPI-overexpressing EA.hy926 cells were counted on Days 3, 5, and 7 to determine the growth curve; (**D**) The EA.hy926 cell morphology was determined by staining with Tetramethylrhodamine (TRITC)-conjugated Phalloidin and DAPI and visualization under a fluorescence microscope; (**E**) The migration ability was determined by wound scratch assay at 0 h and 24 h; (**F**) The relative migration calculated from the wound scratch assay. Each bar represents the mean ± SEM. * *p* < 0.05 vs. the EA-WT group (*t*-test, *n* = 3).

The EA.hy926 cell line has less basal SLPI expression (Figure 2A); therefore, overexpression of SLPI in endothelial cell line EA.hy926 was performed by exogenous gene transfection. Four clones E3, F5, G3, and G5 were successfully selected by limiting dilution and were cultured in selective medium containing 100 µg/mL of hygromycin B (data not shown). Stable cell lines overexpressing rhSLPI were generated from a single colony of transfected cells until the appropriate amount for the experiment was reached. Expression of rhSLPI proteins was measured from collected conditioned medium by ELISA and compared to the wild type. The results showed that the G3 clone secreted the highest level of rhSLPI compared to the others (data not shown), and this was used in further experiments. Expression of rhSLPI in the stable cell line overexpressing rhSLPI was significantly higher than that of wild-type EA.hy926 cells (1378 ± 77.80 pg/mL and less than 25 pg/mL, *p* < 0.05) (Figure 2A).

After establishing that EA.hy926 cells were able to overexpress SLPI (EA-SLPI), biological characteristics such as their growth curve, population doubling time (PDT), and cell morphology were measured and compared to wild-type EA.hy926 cells (EA-WT). The cell proliferation of EA-WT cells and EA-SLPI cells was similar when determined by the growth curve (Figure 2B). The results showed that the PDTs of these cell lines were not significantly different (Figure 2C), while the cell morphologies of EA-WT cells and EA-SLPI cells were similar in terms of size and shape. However, the determination of cytoskeleton organization by Phalloidin–TRITC staining showed minor differences in EA-SLPI cells by increased actin filament density in the margins of the cells (white arrow) compared to the wild type (Figure 2D). Cell migration is one of the characteristics of endothelial cells, so cell migration ability was determined by wound healing assay. The results showed that EA-SLPI cells (59.56% ± 5.59%, *p* < 0.05) were not significantly different when compared to EA-WT cells (66.87% ± 3.78%, *p* < 0.05) (Figure 2E,F).

### 3.2. Overexpression of SLPI Reduced Vascular Endothelial Cell Injury Caused by In Vitro Simulated Ischemia/Reperfusion (sI/R)

**Figure 3:**
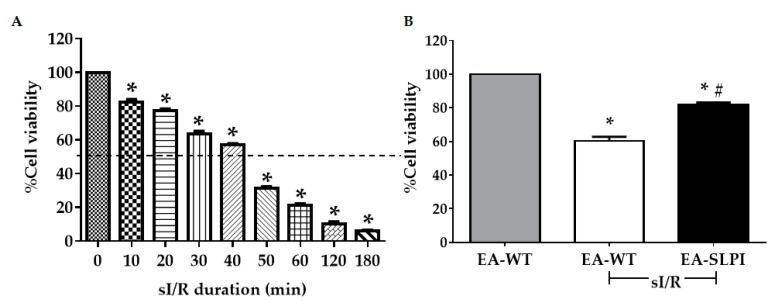
Determination of SLPI-overexpressing EA.hy926 cells in ischemia/reperfusion (I/R). (**A**) EA.hy926 cells were subjected to 10–180 min simulated ischemia (sI) followed by 24 h of reperfusion; (**B**) Wild-type (EA-WT) and SLPI-overexpressing (EA-SLPI) EA.hy926 cells were exposed to 40 min of sI followed by 24 h reperfusion, and the cell viability was determined by MTT. Each bar represents the mean ± SEM. * *p* < 0.05 vs. non-sI/R-treated group (ANOVA), # *p* < 0.05 vs. the sI/R-treated group (ANOVA, *n* = 3).

After a stable endothelial cell line overexpressing SLPI was established, the protective effect of rhSLPI during in vitro simulated ischemia/reperfusion (sI/R) on endothelial cells was determined. Before the simulated ischemia/reperfusion (sI/R) experiment was performed, the duration of sI/R that caused 50% cell death was optimized. Cells were exposed to sI buffer for several periods followed by reperfusion for 24 h. The results showed that simulated ischemia reduced cell viability in a time-dependent manner (Figure 3A). The sI/R at 40 min significantly reduced cell viability by approximately ~50% (57.09% ± 0.78%, *p* < 0.05). Therefore, this time point was used in the sI/R protocol for further experiments.

Then, both EA-WT cells and EA-SLPI cells were subjected to 40 min of sI buffer followed by 24 h of reperfusion. The relative cell viability was determined by MTT assay. The results showed that sI/R reduced the relative percentages of cell viability in EA-WT cells (60.27% ± 2.52%). Overexpression of SLPI in EA.hy926 (EA-SLPI) cells significantly increased cell viability when compared to EA-WT (81.75% ± 1.42%, *p* < 0.05) (Figure 3B).

### 3.3. Overexpression of SLPI Reduced Hypoxia/Reoxygenation-Induced Vascular Endothelial Cellular Injury

**Figure 4:**
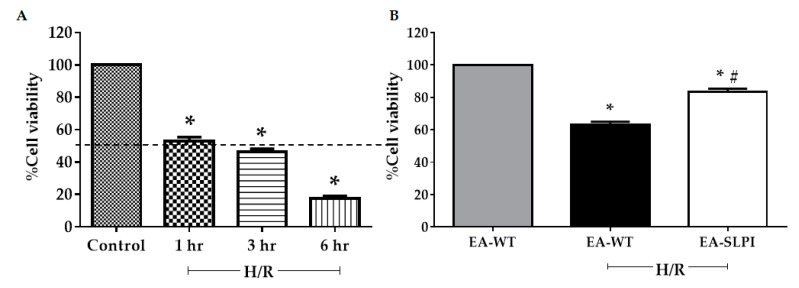
Determination of SLPI-overexpressing EA.hy926 cells in hypoxia/reoxygenation (H/R). (**A**) EA.hy926 cells were subjected to 1, 3, and 6 h hypoxia followed by 3 h of reoxygenation; (**B**) Wild-type (EA-WT) and SLPI-overexpressing (EA-SLPI) EA.hy926 cells were exposed to 1 h of hypoxia followed by 3 h reoxygenation, and the cell viability was determined by MTT. Each bar represents the mean ± SEM. * *p* < 0.05 vs. non-H/R-treated group (ANOVA), # *p* < 0.05 vs. H/R-treated group (ANOVA, *n* = 3).

EA-SLPI cells can produce and secrete rhSLPI to the culture medium. Here, the cytoprotective effect of secreted rhSLPI on themselves and other cardiac neighboring cells, in this case, H9c2 cardiomyocytes, was determined. However, the in vitro sI/R model required the removal of the culture medium and replacement with sI buffer to wash out the effect of rhSLPI during sI/R injury. Therefore, a hypoxia/reoxygenation (H/R) model was used instead of sI/R. Optimization of the H/R duration that caused 50% cell death was performed by overlaying a liquid paraffin cover on the culture medium surface (hypoxia) for several time periods, followed by reoxygenation for 3 h. The results demonstrated that hypoxia reduced cell viability in a time-dependent manner. Hypoxia at 1 h significantly reduced cell viability to ~50% (52.99% ± 2.47%, *p* < 0.05) when compared to the control (Figure 4A). Thus, H/R at 1 h/3 h durations was used in the H/R protocol in further experiments.

After H/R optimization, both EA-WT cells and EA-SLPI cells were exposed to 1H/3R. At the end of reoxygenation, cell viability was determined by MTT assay. The results showed that H/R reduced the relative percentages of cell viability in EA-WT cells when compared to the control group (63.07% ± 1.93%, *p* < 0.05). Overexpression of SLPI in EA-SLPI cells significantly increased cell viability when compared to EA-WT cells (83.57% ± 1.78%, *p* < 0.05) (Figure 4B).

### 3.4. Overexpression of SLPI Reduced sI/R-Induced Intracellular ROS Production in Vascular Endothelial Cells

**Figure 5:**
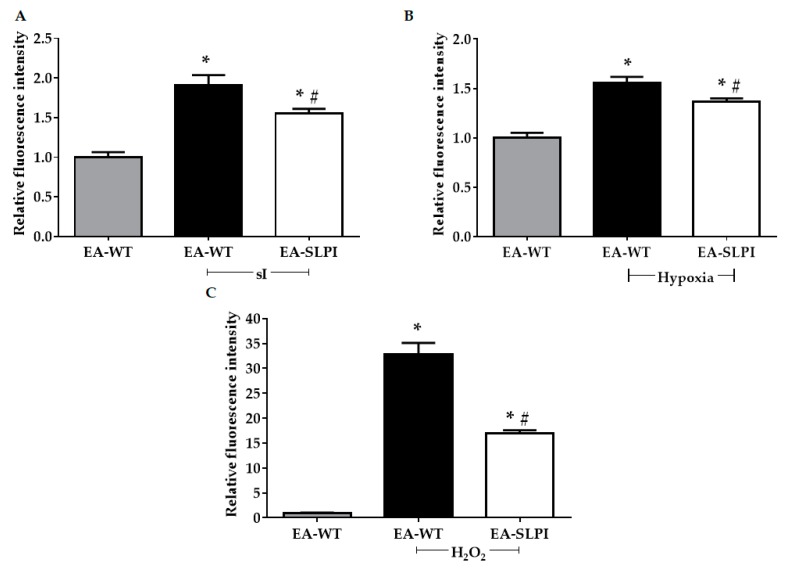
Determination of intracellular reactive oxygen species (ROS) levels in SLPI-overexpressing EA.hy926 cells. Wild-type (EA-WT) and SLPI-overexpressing (EA-SLPI) EA.hy926 cells were exposed to H2DCFDA followed by (**A**) sI/R or (**B**) H/R or (**C**) H_2_O_2_ challenge. Then, the intracellular ROS level was determined by spectrophotometry. Each bar represents the mean ± SEM. * *p* < 0.05 vs. nontreated group (ANOVA), # *p* < 0.05 vs. treated group (ANOVA, *n* = 3).

Previous studies reported that SLPI acts as an ROS scavenger by reducing intracellular ROS production in many cells during sI/R [11,12,13,14]. To prove that SLPI secreted from EA-SLPI cells reduced intracellular ROS production, in vitro intracellular ROS production was determined under simulated ischemia (sI), hypoxia, and H_2_O_2_. The results showed that sI significantly increased the relative intracellular ROS level. Overexpression of SLPI significantly reduced the intracellular ROS level when compared to EA-WT cells (1.91 ± 0.12 vs. 1.55 ± 0.06, *p* < 0.05) (Figure 5A). Hypoxia significantly increased the relative intracellular ROS level, but this was significantly reduced in EA-SLPI cells (1.55 ± 0.06 vs. 1.37 ± 0.03, *p* < 0.05) (Figure 5B). Similar to the findings from the sI and hypoxia experiments, exposure to H_2_O_2_ significantly increased the intracellular ROS level, but this was significantly reduced in EA-SLPI cells (32.85 ± 2.28 vs. 17.01 ± 0.56, *p* < 0.05) (Figure 5C).

### 3.5. Endothelial-Derived rhSLPI Protected Cardiomyocytes from Hypoxia/Reoxygenation-Induced Cell Injury

**Figure 6:**
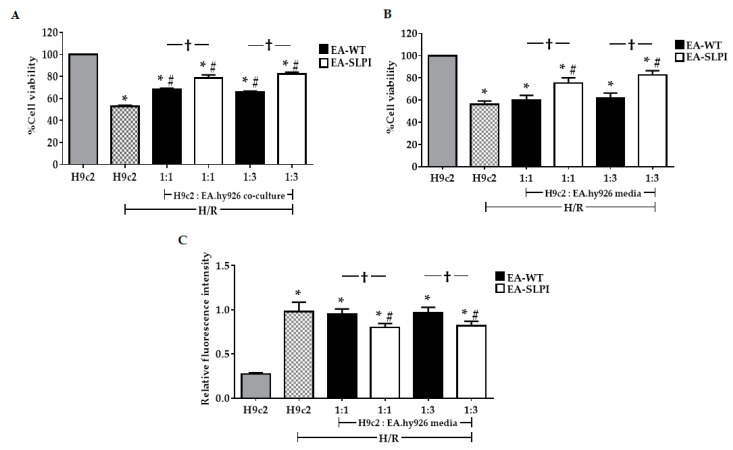
The effect of rhSLPI secreted from the endothelial cell line protected the cardiomyocyte cell line from hypoxia/reoxygenation. (**A**) Wild-type (EA-WT) and SLPI-overexpressing (EA-SLPI) EA.hy926 cells were co-cultured with the H9c2 cell line at ratios 1:1 and 1:3, then subjected to H/R, and the cell viability was determined by MTT; (**B**) The culture media from wild-type (EA-WT) and SLPI-overexpressing (EA-SLPI) EA.hy926 cells were transferred to culture H9c2 cells at ratios of 1:1 and 1:3; then, the cells were exposed to H/R and the cell viability was determined by MTT. After transferring the media from wild-type (EA-WT) and SLPI-overexpressing (EA-SLPI) EA.hy926 cells at ratios of 1:1 and 1:3 to the H9c2 cells, (**C**) the cells were exposed to H2DCFDA followed by H/R, and the intracellular ROS levels were determined by spectrophotometry. Each bar represents the mean ± SEM. * *p* < 0.05 vs. the H9c2 not subjected to H/R (ANOVA), # *p* < 0.05 vs. the H9c2 subjected to H/R (ANOVA), † *p* < 0.05 vs. the 1:1 or 1:3 culture ratio group (ANOVA, *n* =3).

To investigate the cardio-vasculoprotective effect of endothelial-derived rhSLPI, wild-type (EA-WT) and SLPI-overexpressing EA.hy926 cells (EA-SLPI) were indirectly co-cultured, using the Transwell culture system, with rat cardiac myoblast H9c2 cells at initial EC/CM cell density ratios of 1:1 and 3:1. The cells were subjected to 1 h of hypoxia followed by 3 h of reoxygenation. The results showed that H/R significantly reduced H9c2 cell viability when compared to the control (52.82% ± 0.96% vs. 100.00%, *p* < 0.05). Co-culture of H9c2 with EA-WT cells showed significantly higher cell viability than H9c2 alone in both CM/EC ratios of 1:1 and 1:3 (CM/EC 1:1, 67.95% ± 1.27% vs. 52.82% ± 0.96%, *p* < 0.05) (CM/EC 1:3, 65.47% ± 1.22% vs. 52.82% ± 0.96%, *p* < 0.05) (Figure 6A). Interestingly, cardiac cells co-cultured with EA-SLPI cells showed greater cytoprotective effects against H/R injury than did those co-cultured with EA-WT cells at both cell density ratios. The results also indicated that co-culture between H9c2 and EA-SLPI at a ratio of 1:1 enhanced the percentage of cell viability by 10.51% when compared to EA-WT (78.46% ± 2.90% vs. 67.95% ± 1.27%; Δ = 10.51%). At a ratio of 1:3, co-culture between H9c2 and EA-SLPI enhanced the percentage of cell viability by 16.7% when compared to EA-WT (82.17% ± 1.63% vs. 65.47% ± 1.22%; Δ = 16.7%).

The paracrine effect of endothelial-derived SLPI was hypothesized to be through the effect of secreted rhSLPI from EA-SLPI cells. To confirm that endothelial-derived SLPI from ECs provided the cytoprotective factors reducing CM injury, conditioned media from EA-WT cells and EA-SLPI cells with initial seeding density similar to the co-culture experiments at ratios 1:1 and 1:3 were collected and used to treat H9c2 cells before exposure to H/R. The results showed that treatment with conditioned medium from EA-SLPI cells in both original density ratios significantly increased the cell viability when compared to H9c2 cultured in conditioned medium from EA-WT cells (1:1, 75.42% ± 4.74% vs. 60.17% ± 4.09%, *p* < 0.05; 1:3, 82.43% ± 4.06% vs. 61.98% ± 4.34%, *p* < 0.05) (Figure 6B). The results also showed that conditioned medium from EA-SLPI at a CM/EC ratio of 1:1 enhanced the percentage cell viability of H9c2 by 15.25% (75.42% ± 4.74% vs. 60.17% ± 4.09%; Δ = 15.25%). Likewise, culture medium from SLPI-EA at a CM/EC ratio of 1:3 enhanced the percentage of cell viability of H9c2 by 20.45% (82.43% ± 4.06% vs. 61.98% ± 4.34%; Δ = 20.45%).

The effect of rhSLPI from the conditioned media on intracellular ROS production was also determined. The results showed that H9c2 cells cultured in conditioned medium from EA-SLPI cells with initial seeding density similar to the co-culture experiments at ratios 1:1 and 1:3 had significantly decreased relative intracellular ROS levels when compared to H9c2 cultured in conditioned medium from EA-WT cells (1:1, 0.95 ± 0.06 vs. 0.80 ± 0.05, *p* < 0.05; 1:3, 0.97 ± 0.05 vs. 0.82 ± 0.05, *p* < 0.05) (Figure 6C).

### 3.6. Endothelial-Derived rhSLPI Protected Cardiomyocytes from Hypoxia/Reoxygenation Injury via the Akt and p38MAPK Signaling Pathway

**Figure 7:**
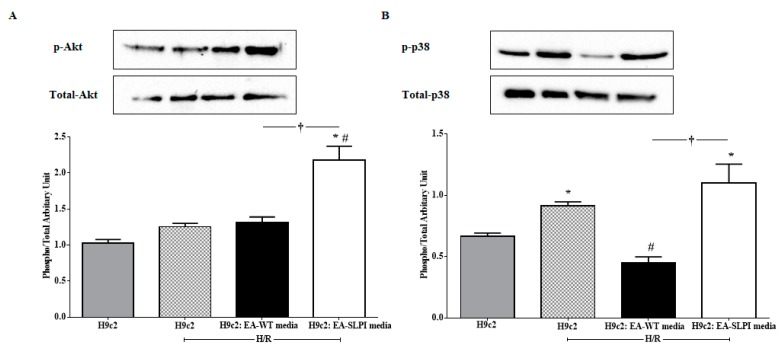
Determination of the effect of rhSLPI secreted from endothelial cells on the cardiomyocyte cell line subjected to H/R in terms of the cellular signaling response. The media from cultured wild-type (EA-WT) and SLPI-overexpressing (EA-SLPI) EA.hy926 cells were transferred to culture H9c2 cells; then, the cells were exposed to H/R, and the activation of Akt (**A**) and p-38 MAPK (**B**) was determined by Western blot analysis. Each bar represents the phosphorylation ratio (phospho/total) of Akt and p38 MAPK. *p < 0.05 vs. H9c2 not subjected to H/R (ANOVA), # *p* < 0.05 vs. H9c2 subjected to H/R (ANOVA), †*p* < 0.05 vs. the H9c2/EA-WT medium group (ANOVA, *n* = 3).

To determine the effect of rhSLPI in cellular signaling, H9c2 cells were treated with conditioned media from EA-WT cells and EA-SLPI cells at an initial seeding density of 1:3 before exposure to H/R. The results showed that treatment with a cultured medium from EA-SLPI cells significantly increased Akt phosphorylation when compared to the control and to H9c2 treated with conditioned medium from EA-WT cells (Figure 7A). Moreover, H/R activated p38 MAPK phosphorylation. Treatment with conditioned medium from EA-WT cells significantly reduced p38 MAPK phosphorylation in H9c2 cells. By contrast, treatment with cultured medium from EA-SLPI significantly increased p38 MAPK phosphorylation when compared to culture in the medium from EA-WT (Figure 7B).

### 3.7. Endothelial-Derived rhSLPI Reduced the Apoptotic Regulatory Signaling Protein Level

**Figure 8:**
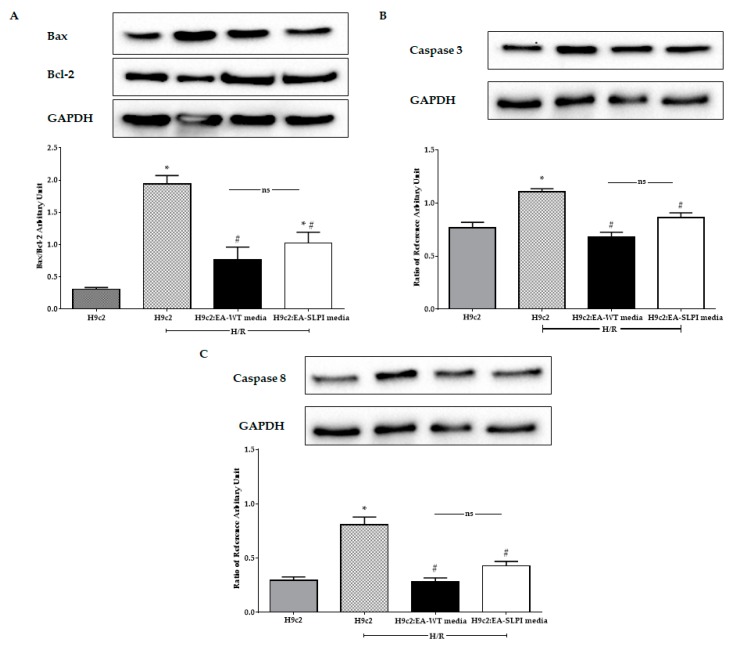
Determination of the effect of rhSLPI secreted from endothelial cells on the cardiomyocyte cell line subjected to H/R in terms of the apoptosis pathway. The media from cultured wild-type (EA-WT) and SLPI-overexpressing (EA-SLPI) EA.hy926 cells were transferred to culture H9c2 cells; then, the cells were exposed to H/R, and the apoptotic proteins, including Bax/Bcl-2 (**A**), caspase-3 (**B**), and caspase-8 (**C**), were determined by Western blot analysis. Each bar represents the ratio relative to the reference protein (GAPDH). *p < 0.05 vs. H9c2 not subjected to H/R (ANOVA), # *p* < 0.05 vs. H9c2 subjected to H/R (ANOVA, *n* = 3).

H/R increased the Bax/Bcl-2 ratio in H9c2 when compared to the control. By contrast, treatment with cultured medium from EA-WT cells and EA-SLPI cells significantly decreased the Bax/Bcl-2 ratio when compared to untreated H9c2 (Figure 8A). Furthermore, H/R also increased the expression of apoptotic regulatory enzymes caspase-3 and caspase-8 in H9c2 when compared to the control group (Figure 8B,C). Treatment with conditioned media from both EA-WT cells and EA-SLPI cells significantly reduced the expression of caspase-3 and caspase-8. Treatment of H9c2 with conditioned medium from EA-WT cells showed a greater reduction in the expression of caspase-3 and caspase-8, but the results were not significantly different from those for the EA-SLPI group.

## 4. Discussion

**Figure 9:**
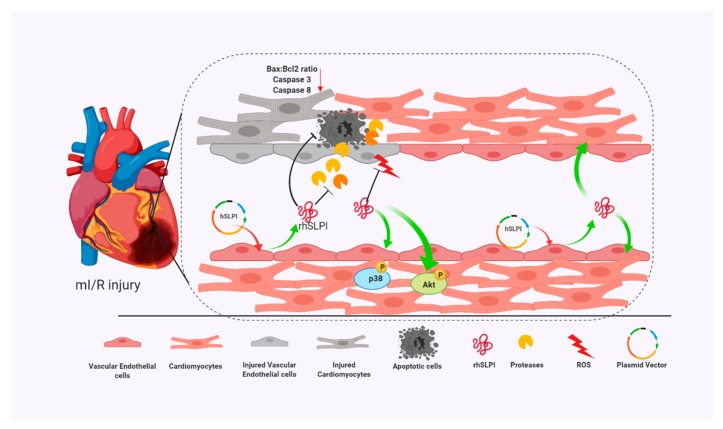
A schematic diagram of the major findings in this study. The rhSLPI that was overexpressed and secreted from vascular endothelial cells could protect not only endothelial cells but also cardiomyocytes from I/R injury. The mechanisms for this could be due to the attenuation of intracellular ROS production, activation of Akt and p38 MAPK phosphorylation, and reduction of apoptotic regulatory proteins (Bcl-2, caspase-3, caspase-8).

The cytoprotective effect of secretory leukocyte protease inhibitor (SLPI) has been reported in different cardiac resident cells, including cardiomyocytes, cardiac fibroblasts, and vascular endothelial cells [11,13,14]. Moreover, SLPI has also been shown to provide cardioprotection against I/R injury [10,12]. Here, using an in vitro genetic manipulation technique, we provided evidence that endothelial-derived recombinant human secretory leukocyte protease inhibitor (rhSLPI) protects cardiac cells from ischemia/reperfusion (I/R) injury via paracrine signaling. The major findings from this study are that in vitro overexpression of human SLPI cDNA in vascular endothelial cells (ECs) reduced injury to themselves in I/R and H/R via the attenuation of intracellular ROS production. EC-derived rhSLPI protected against H/R-induced cardiomyocyte death via the reduction of intracellular ROS production, the activation of pro-survival kinase Akt phosphorylation, pre-conditioned p38 MAPK phosphorylation, and the attenuation of apoptotic regulatory molecules, Bax/Bcl-2, caspase-3, and caspase-8 expression (Figure 9).

Endothelial-derived factors have been studied for many decades. Several have been well documented as well-recognized key regulators for vascular homeostases, such as endothelial-derived relaxing factor (EDRF)—nitric oxide (NO), prostacyclin (PGI2), and endothelial-derived hyperpolarizing factor (EDHF) [17]. The implementation of complementary genetic and pharmacological approaches provided an explanation of the physiological and biochemical roles of endothelial-derived factors, such as regulating vascular integrity, controlling smooth muscle tone, and stimulating circulation of leukocytes and platelets [18]. The roles of endothelial-derived factors are not only limited to blood vessels but are also crucial for the heart, especially via paracrine signaling. This phenomenon has been termed as endothelial–cardiomyocyte (EC–CM) crosstalk [19] and is regarded as essential for normal cardiac development as well as normal pathological conditions, including ischemia, remodeling, and metabolic dysfunction [19]. A more important role of EC–CM crosstalk that enhanced pharmacological cardioprotection was highlighted by Leucker et al. [20]. Therefore, manipulation of EC–CM crosstalk, especially by novel EC-derived cardioprotective factors, is a possible alternative therapeutic strategy to reduce myocardial I/R injury. The current study hypothesized that endothelial-derived rhSLPI could prime the cardiomyocytes and enhance the cardioprotective effect on cardiac cells subjected to in vitro I/R injury. The findings from the current study showed that endothelial-derived rhSLPI showed a greater protective effect than that found using recombinant human SLPI in our previous studies on cardiac resident cells [11,12,13,14].

Molecular crosstalk between ECs and CMs is conducted using bidirectional signals. CMs secrete factors that contribute to vasculature growth, while ECs promote the physiological and structural maturation of CMs [21]. Genetic manipulation of ECs to produce and secrete cytoprotective factors may protect CMs from injury and vice versa. However, in vivo cardiomyocyte overexpression of genes of interest is difficult due to the efficiency of gene transfer [22,23] and may interfere with the electrophysiological properties of the heart [11], possibly leading to abnormal functioning. Overexpression of genes of interest in other cardiac neighboring cells, particularly endothelial cells, may be more suitable. The secretory leukocyte protease inhibitor (SLPI) is known to be secreted in response to inflammation, primarily in mucosal tissue [9]. There was no information regarding gene expression or SLPI secretion from the vasculature, either to the blood stream or at basal sites; this correlated with our findings that the vascular endothelial cell line EA.hy926 showed a low SLPI expression level. Generation of a stable cell line overexpressing rhSLPI was established in the stable EA.hy926 cell line. This showed greater rhSLPI production without changing the biological properties of the ECs when compared to the parental cell line. Other endothelial cell functions such as the secretion of von Willebrand factor (vWF), nitric oxide (NO), and 6-keto-prostaglandin F1α (a metabolite of prostacyclin) require further investigation.

Overexpression of rhSLPI in vascular endothelial cells reduced sI/R- and H/R-induced cell death by reducing intracellular ROS production. This finding concurred with a previous study reporting that pre-treatment of rhSLPI reduced sI/R-induced HUVEC death and injury [13]. Interestingly, endothelial-derived rhSLPI in this study provided superior cytoprotective effects against cardiac cell death than did pre-treatment recombinant protein. Our previous findings determined the effective concentration of rhSLPI for direct treatment in microgram levels [13,14]; however, here, endothelial-derived rhSLPI was secreted in nanogram levels. The beneficial effect might not only be due to rhSLPI but may also require other endothelial-derived paracrine factors. Crosstalk between ECs and CMs is essential for cell survival. In a physiological heart, the ratio of ECs to CMs is known to be approximately 3:1 [19]. A previous study also reported that co-culture of ECs with CMs at a ratio of 3:1 significantly decreased H/R-induced CM injury [6]. Our results support that EC and CM crosstalk is essential for cell survival (Figure 6A). Although the actual effect of ECs as protectors of CMs during ischemic injury is still controversial, the available evidence highlights this possibility. A variety of endothelial-derived factors have been proposed to be secreted from ischemic ECs, such as neuregulin (NRG)-1 β and endothelial exosome. Exposure to ROS induced NRG-1 release from endothelial cells to protect isolated adult rat ventricular myocytes (ARVMs) from I/R-induced apoptosis [24]. A recent report also demonstrated that endothelial-derived exosomes conferred resistance to sIR injury in cardiomyocytes, and this may contribute to ischemic preconditioning (IPC) [25].

Here, the paracrine effect of endothelial-derived rhSLPI was proved by an in vitro assay via indirect co-culture using a Transwell chamber technique which has been used in several cardiac cell studies [26,27]. Both the co-culture experiments and the experiments using conditioned medium also suggested the paracrine effect of endothelial-derived rhSLPI. Moreover, the paracrine effect of endothelial-derived rhSLPI is believed to play a cardioprotective role via the preconditioning of cardiac cells. Ischemic preconditioning (IPC) is considered to be any intervention that generates tolerance towards subsequent ischemia. The heart could also be preconditioned by chemicals as well as by mechanical IPC, referred to as “chemical preconditioning”. Several signal transduction pathways have been elucidated to involve preconditioning, such as PI3-kinase and Akt, protein kinase C, and protein kinase A-p38 MAPK [28].

In this study, the intracellular mechanism of endothelial-derived SLPI against I/R-induced cardiomyocyte injury was demonstrated in two major signaling molecules: Akt survival kinase and p38 MAPK activation. The results showed that SLPI secreted from endothelial cells predominantly activated Akt survival kinase (Figure 7A), which correlated well with the cell survival (Figure 6B). This result could be clearly interpreted as SLPI activating Akt survival kinase and saving cardiac cells from injury. However, the interpretation of p38 MAPK is more complicated.

Myocardial I/R injury induces cell death via several signaling pathways, predominantly p38 MAPK [29,30]. It is well known that ischemia could activate p38 MAPK, which subsequently leads to cell death [31,32]. It can be clearly seen in Figure 7B (second bar) that H/R could activate p38 MAPK in H9c2 cells, which correlated to cell survival, reduced in H/R (Figure 6B). As mentioned in the introduction, the release of paracrine factors from ECs has been known to contribute to pharmacological cardioprotection [20], which can be clearly seen in the results in Figure 7B (third bar), where conditioned medium from EC-WT reduced p38 MAPK activation and was related to cell survival (Figure 6B). It is noteworthy that p38 MAPK is involved in innumerable biological processes and therefore, not surprisingly, under many circumstances its activation leads to myocardial protection rather than to injury [33,34,35,36]. Not only mechanical ischemic preconditioning (IPC) but also pharmacological preconditioning [37,38] could activate p38 MAPK and reduce cardiac cell death. In this study, endothelial-derived SLPI was able to significantly reduce cardiac cell death, while activation of p38 MAPK was observed in cells treated with conditioned medium from EA-SLPI (Figure 7B). This could possibly be interpreted as endothelial-derived SLPI protecting cardiac cells from I/R injury via the pharmacological preconditioning concept. All in all, our study suggests that endothelial-derived SLPI can activate Akt survival kinase and preconditioning by the activation of p38 MAPK, which plays a critical role in the crosstalk between cardiomyocytes and the vasculature [39]. However, the actual signal pathways that are involved in the protective effect of SLPI should be further intensively investigated by using specific inhibitors for each possible signaling pathway.

There were some limitations of this study in the use of the vascular endothelial cell line (EA.hy926) as a model of study. For more physiologically relevant and clinical applications, primary cells should be used in this study. Primary vascular endothelial cells, such as HUVECs, are ideal for studying the biology of vascular endothelial cells. However, there are several points that need to be considered for their use in the current experimental design. Firstly, HUVECs have a relatively short life span in vitro, and their phenotype can change with successive passages. Secondly, our study aimed to produce stable cells overexpressing SLPI by stable transfection, but it is known that HUVECs can be difficult to transfect, and their finite life span makes it problematic to develop stably transfected cell lines. As Seidl et al. suggested, to overcome the problems of using primary endothelial cells, immortalized endothelial cell lines have been developed [40]

Another limitation of this study was using in vitro experimentation as a model, which may not represent real physiology. However, there are several benefits of an in vitro experimental design. For instance, an in vitro model allows precise environmental settings, especially for hypoxia and reoxygenation on cardiomyocytes/vascular endothelial cells without confounding influences by factors such as other non-cardiomyocyte populations or circulating factors such as hormones, neurotransmitters, and cytokines [41]. Therefore, an in vitro model was best suited to investigate the direct effect of therapeutic agents (in this case, rhSLPI) on both cardiomyocytes and vascular endothelial cells and also their cellular crosstalk. The application of knowledge gained from this in vitro study will provide basic information for further in vivo studies, for example, in vivo vascular specific overexpression of rhSLPI in an animal model subjected to I/R injury, cardiac remodeling, and heart failure. In addition, as it is known that the main function of SLPI is anti-inflammation, the effect of SLPI on cardiac inflammatory cytokine levels or leukocyte infiltration in infarct heart tissue still needs to be investigated. This could benefit inflammation-related cardio-vasculopathies such as atherosclerosis, infective cardiomyopathy, inflammatory cardiomyopathy, etc. Therefore, future work could be focused on elucidation of the effect of endothelial-derived rhSLPI on inflammation-related cardio-vasculopathies by determining the inflammatory cytokine levels and the activation of cardiac resident leukocytes or macrophages. Furthermore, an interesting application for rhSLPI in real clinical settings could be the implementation of rhSLPI as a novel or alternative additive compound to improve the quality of isolated graft tissue, especially vessel or heart, by adding it to the organ preservative solution for transplantation.

## 5. Conclusions

This is the first study to demonstrate that manipulation of ECs to secrete rhSLPI provides cardio-vasculoprotection against I/R and H/R injury. Preconditioning of endothelial-derived rhSLPI via activation of Akt and p38 MAPK has the potential to reduce both CM and EC death by the attenuation of intracellular ROS production, Bax/Bcl-2 ratio, caspase-3, and caspase-8.
